# Isotopic paleoecology of Northern Great Plains bison during the Holocene

**DOI:** 10.1038/s41598-019-52873-4

**Published:** 2019-11-12

**Authors:** Gaimi Davies, Blake McCann, Jay Sturdevant, Fern Swenson, Igor V. Ovchinnikov

**Affiliations:** 1 Department of Biology, University of North Dakota, Grand Forks, ND USA; 2 Resource Management, Theodore Roosevelt National Park, Medora, ND USA; 3 Midwest Archaeological Center, National Park Service, Lincoln, NE USA; 4 Archaeology & Historic Preservation Division, State Historical Society of North Dakota, Bismarck, ND USA; 5 Forensic Science Program, University of North Dakota, Grand Forks, ND USA

**Keywords:** Conservation biology, Palaeoecology, Stable isotope analysis, Palaeoecology

## Abstract

Bison (*Bison bison*) are one of the few terrestrial megafauna to survive the transition into the Holocene and provide a unique opportunity to study a species on a broad spatiotemporal scale. Today, bison are primarily managed in small and isolated herds with little known about their ancestral ecology. We studied the carbon and nitrogen isotopes of Northern Great Plains bison from the terminal Pleistocene and throughout the Holocene to gain insight into their paleoecology. This time span is contemporary with the first population bottleneck experienced by bison at the end of the Pleistocene and includes the second bottleneck which occurred in the late 19^th^ century. Results were compared with modern bison herd isotopic values from Theodore Roosevelt National Park (TRNP). Patterns of isotopic variation found in bison over time indicate significant (δ^13^C p = 0.0008, δ^15^N p = 0.002) differences in diet composition and correlate with climate throughout the Holocene. Isotopic relationships described here reveal the plasticity of ancient bison in unrestricted rangelands during periods of climatic fluctuations. Managers at TRNP and elsewhere should pursue opportunities to expand bison range to maximize forage opportunities for the species in the face of future environmental change.

## Introduction

The transition from the Pleistocene into the Holocene epoch (~11.7 thousand years ago) marked the disappearance of many North American megafauna. As this mass extinction event is more thoroughly investigated it has become clear that the cause cannot be attributed to any singular explanation for all species or regions^1–4^. Bison (*Bison bison*) are one of the few terrestrial megafauna to survive the late Pleistocene extinction in North America. Because of their longevity on the landscape and widespread historical range, they provide a unique opportunity for species study across broad temporal and spatial scales.

Bison entered North America from Eurasia via the Bering Land Bridge during two separate windows of time (195 to 135 thousand years ago and 45 to 21 thousand years ago) when the area’s ice sheets retreated and the exposed ground was above sea level^5^. They rapidly colonized lower latitudes of North America when an ice-free corridor between the Cordilleran and Laurentide ice sheets opened around 14 to 13.5 thousand years ago^2,6^. In North America, the stable climate during interglaciation led to the rapid spreading of peatlands and dense forests, limiting connectivity of suitable habitat and the ability of megafauna to disperse when coping with climate change^2,7^. This issue was confounded by rising sea levels that flooded former dispersal corridors^2,4^. Fossil records show that animal populations were dwindling before evidence of human presence even though some researchers have attributed the loss of Pleistocene megafauna at least in part to human activity^2,4^.

Ancient DNA data reveal that bison experienced a drastic reduction in numbers during the terminal Pleistocene, resulting in a genetic bottleneck^8,9^. Subsequently, they successfully repopulated North America in the tens of millions by the Early Holocene, spanning from Alaska to Mexico^6,9^. Near the end of the 19^th^ century, bison suffered a second bottleneck when they were reduced to a few hundred individuals as a result of European settlement of the continent^9,10^. Through conservation efforts enacted during the last century, North American bison have been brought back from the brink of extinction, but their long-term viability as a species remains threatened due to restricted rangelands, artificial selection within confined herds, and a lack of gene flow between herds. Questions remain about the genetic diversity currently found in conservation herds and how the species will respond to environmental change within restricted areas. Analysis of stable isotopes in bison remains may help shed light on bison diet and foraging habitat selection over time, providing insights regarding physiological plasticity of the species relevant to management.

Isotopic biogeochemistry of collagen found in ancient bones and teeth is increasingly used in the construction of paleoecology and paleoenvironments. In addition to recording climatic variables such as temperature and precipitation, stable isotopic signatures encapsulate feeding strategies of animals from the past^11–13^. Isotopic ratios of carbon (δ^13^C) and nitrogen (δ^15^N) are assimilated into herbivore skeletal collagen and tooth dentin through diet, recording the isotopic composition of plant material consumed^14^. These values will remain the same over time in well-preserved specimens^15^. Bone collagen has a slow turnover rate and will record several years of the animal’s life^16–18^. Tooth dentin forms at a specific point in development and records a shorter window of time, in the order of months^19,20^. The carbon isotopic signature depends on the proportion of plants having C_3_ and C_4_ photosynthetic pathways in ecosystems^21,22^ as well as changes in atmospheric CO_2_ from canopy cover in forested areas^23^ or post-industrial revolution CO_2_ emissions^13^. The values of C_3_ plants in North America range from −30 to −24‰ and C_4_ plants fall between −15 and −11‰^24^. This allows us to distinguish between grazers, browsers, and mixed feeders due to the depletion in ^13^C (i.e. lower δ^13^C values) observed in grazing diets^17^. Bison exhibit an enrichment factor of 6.3‰^25^ when carbon isotopes are assimilated into their skeletal tissue and this value needs to be factored into calculating the percentage of C_3_ and C_4_ plants in bison diet. Therefore, the expected δ^13^C values of bison feeding primarily on C_3_ plants would be between −23.7 and −17.7‰, while C_4_ bison diets would range from −8.7 and −4.7‰. Throughout the Holocene, the Great Plains have been largely dominated by *Poaceae* (grass) communities^26^, which can exhibit C_3_ or C_4_ photosynthetic pathways. The climate in the Northern Great Plains has predominately favored the C_3_ subfamily *Pooideae* with a smaller amount of C_4_ subfamilies, *Panicoideae and Chloridoideae*. The abundance of C_4_ grasses increases as warm seasons get longer, allowing us to capture climatic changes in the diets of grazers^22^. Nitrogen isotopic values provide insight into moisture level and nutritional stress due to an observed increase in δ^15^N in animal tissue from the recycling of urea under conditions of drought^15,17,27,28^. However, there are other factors that contribute to nitrogen values in herbivores. Higher nitrogen can indicate warmer temperatures and a diet composed of more graminoids and herbs than trees and shrubs^13,29–32^. In modern European bison, it was found that canopy cover had the biggest influence on δ^15^N, where less light will decrease nitrogen values in plants^29^.

North American isotopic studies of bison to date have primarily focused on Pleistocene paleoecology^30,31,33–36^, climatic interpretations^11,32^, and values from modern herds^12,27,37^. At present, there are few isotopic studies of Holocene bison in North America. Existing research covers relatively short time periods or small sample sizes^11,38–41^. The limited data on North American bison from the Holocene may be in part due to the prior perception of a relatively stable climate during this epoch, though the most recent studies of paleoclimate portray the Holocene as a dynamic period with fluctuations in temperature and precipitation^42–44^. An analysis of approximately fifty paleoclimatic records of greenhouse gases, glacial coverage, and pollen profiles determined that the Holocene had several periods of sudden climate change outlined by variations in atmospheric circulation, moisture, and temperature changes^43^. For instance, a study of pollen records found three periods in which climate rapidly heated during the Holocene^43^ and evidence of a steep drop in temperature at ~8.2 thousand years ago as well as other large fluctuations in temperature throughout the Holocene are supported by the analysis of Greenland ice cores^44^. Therefore, bison in North America during the Holocene had to adapt to a wider range of climatic conditions than previously thought. The amount of variation seen in bison isotopic values throughout specific time periods provides insight into their use of different resources as the environment changed^39,40^.

**Figure 1:**
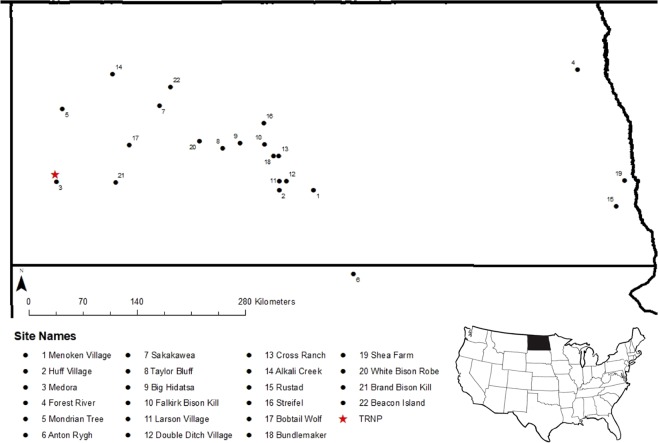
Locations of sample sites within North Dakota and South Dakota, USA. Black circles indicate archaeological sites and a red star identifies the modern bison sample site, Theodore Roosevelt National Park (TRNP). Map layer sourced from: Esri, derived from Tomtom North America, Inc. “U.S. State Boundaries, North Dakota” [downloaded file]. Scale 1:4,750,000. “USA State Boundaries”. URL: https://www.arcgis.com/home/item.html?id = 540003aa59b047d7a1f465f7b1df1950. (September 9, 2019). This map was created using ArcGIS® software version 10.6 by Esri (www.esri.com).

The above-described wealth of information available on Holocene and Pleistocene isotope variation provides a unique opportunity to better understand historic bison through analysis of their bones and teeth represented in archaeological and natural history collections in the Northern Great Plains. Here, we explore a subset of that history through analysis of bison specimens from 22 archaeological sites across the Northern Great Plains spanning the Late Pleistocene to the Late Holocene in comparison to extant bison from Theodore Roosevelt National Park (TRNP), North Dakota, USA (Fig. 1). Our objectives are to: 1) identify δ^13^C and δ^15^N variation indicative of environmental change across time, and 2) utilize isotopic signatures to elucidate feeding ecology of historic bison in context of modern counterparts in North Dakota. We expect that isotopic values in bison will follow changes in climate and subsequently, isotope values of plant material. We expect increased δ^13^C and δ^15^N during periods of warm and dry conditions^22^.

## Results

Fifty-five of the fifty-nine samples of ancient bison bones and teeth yielded sufficient collagen (>1%) to produce reliable data. All samples included in the analysis have C:N ratios between 2.9 and 3.6, relative carbon (%C) >30%, and relative nitrogen (%N) between 11 and 16%, indicative of well-preserved collagen (Table 1)^45,46^. Five modern bison tooth samples (Bison 100–Bison 104) from TRNP herds returned carbon and nitrogen isotopic ratios for comparison with ancient samples.

**Table 1:** Bison sample description including archaeological sites, skeletal component, age in cal BP, and diagnostics for well-preserved collagen isotopic values.

Bison	Sample Site	Skeletal Component	Cal BP Date Range		Median Age (cal BP)	% Collagen	% C	% N	C:N	δ^13^C (‰ VPDB)	δ^15^N (‰ AIR)
1	Medora	molar fragment	270	12	112	4.4	45.0	16.3	3.2	−17.4	6.2
2	Mondrian Tree 32MZ58	bone fragment	3357	3241	3291	4.5	46.9	16.0	3.4	−21.1	8.1
3	Mondrian Tree 32MZ58	phalanx	2724	2497	2580	3.0	42.6	14.9	3.3	−18.5	4.9
4	Anton Rygh 39CA4	metatarsal	501	332	469	6.3	42.9	15.1	3.3	−14.8	5.8
5	Anton Rygh 39CA4	carpal	481	318	388	5.3	45.6	16.7	3.2	−17.5	5.7
6	Menoken Village 32BL2	phalanx	910	787	863	2.6	45.9	16.3	3.3	−19.1	6.4
10	White Bison Robe 32ME7	molar fragment	679	571	665	2.5	44.4	16.5	3.1	−17.8	6.8
11	White Bison Robe 32ME7	molar	460	313	381	4.7	45.9	17.0	3.2	−17.8	6.6
12	Huff Village 32MO11	molar fragment	527	501	513	5.0	46.0	16.8	3.2	−19.3	6.0
13	Huff Village 32MO11	M3	518	487	504	9.9	41.6	15.3	3.2	−18.6	6.5
14	Alkali Creek 32DU336	premolar	1529	1412	1466	3.0	39.8	14.5	3.2	−18.3	6.9
18	Sakakawea 32ME11	M3	302	7	166	5.4	46.4	16.7	3.2	−18.8	6.3
20	Rustad Site 32R1775	molar fragment	8176	8041	8107	1.4	40.2	14.3	3.3	−16.3	9.3
22	Streifel Site 32ML903	head of femur	7834	7701	7768	3.8	41.9	15.0	3.3	−14.6	5.0
25	Big Hidatsa 32ME12	molar	294	14	167	5.9	45.4	16.5	3.2	−18.6	7.4
26	Big Hidatsa 32ME12	M3	290	14	170	4.3	44.0	16.4	3.1	−20.5	7.5
29	Falkirk Bison Kill 32ML927	vertebrae	694	661	677	8.2	43.1	14.9	3.4	−15.6	5.8
30	Falkirk Bison Kill 32ML927	molar	309	156	296	3.5	44.9	16.7	3.1	−16.3	6.4
31	Bobtail Wolf 32DU955A	phalanx	283	5	189	6.2	44.9	15.6	3.4	−20.1	6.1
32	Larson Village 32BL9	M3	491	320	437	3.8	45.3	16.9	3.1	−17.1	5.5
33	Larson Village 32BL9	metatarsal	485	319	429	5.4	43.9	16.0	3.2	−20.3	7.3
34	Double Ditch Village 32BL8	molar fragment	465	315	379	2.5	44.2	16.5	3.1	−15.1	5.0
35	Bundlemaker 32OL159	molar	1173	979	1027	6.2	42.3	15.2	3.2	−16.7	6.6
36	Cross Ranch 32OL151	M3	1175	1010	1107	5.0	44.2	16.1	3.2	−17.2	6.4
37	Taylor Bluff 32ME366	phalanx	272	11	125	6.2	42.3	15.2	3.2	−18.2	5.8
38	Taylor Bluff 32ME366	molar fragment	294	14	167	3.6	44.8	16.4	3.2	−20.1	7.0
39	Falkirk Bison Kill 32ML927	molar fragment	673	566	656	1.5	40.4	14.3	3.3	−19.4	8.3
40	Shea Farm 32CS101	premolar root	527	492	509	5.2	43.0	15.6	3.2	−16.5	5.5
41	White Bison Robe 32ME7	M3	465	315	379	3.6	44.7	16.5	3.2	−19.6	8.3
42	White Bison Robe 32ME7	M3	458	307	386	6.3	42.3	15.5	3.2	−20.3	8.0
43	Menoken Village 32BL2	molar fragment	1175	989	1077	9.7	41.9	15.3	3.2	−15.4	6.2
44	Brand Bison Kill 32SK201	atlas	703	668	683	3.5	45.8	16.5	3.2	−19.7	6.2
45	Brand Bison Kill 32SK201	atlas	688	665	675	2.5	44.8	16.0	3.3	−20.3	7.5
46	Forest River	molar fragment	284	22	185	3.7	44.9	16.4	3.2	−17.8	6.0
47	Falkirk Bison Kill 32ML927	molar fragment	670	565	653	2.8	45.4	16.3	3.3	−20.2	6.9
48	Rustad Site 32R1775	molar	7972	7860	7936	7.2	43.6	15.8	3.2	−17.9	8.8
49	Alkali Creek 32DU336	molar	2750	2539	2728	5.0	42.6	15.8	3.2	−18.3	7.8
50	Rustad Site 32R1775	mandible	9029	8786	8999	2.1	42.0	15.0	3.3	−18.2	6.6
51	Mondrian Tree 32MZ58	M3	631	516	544	12.0	42.5	15.6	3.2	−19.8	6.8
52	Alkali Creek 32DU336	premolar root	1567	1416	1533	5.7	43.0	16.0	3.1	−20.3	7.3
53	Streifel Site 32ML903	bone fragment	4079	3897	3964	11.3	42.0	15.4	3.2	−20.3	7.0
54	Beacon Island 32MN234	molar fragment	12389	12030	12209	1.0	37.5	13.0	3.4	−21.5	6.2
55	Streifel Site 32ML903	metatarsal	4082	3892	3966	12.6	42.0	15.5	3.2	−19.1	6.3
56	Mondrian Tree 32MZ58	bone fragment	2744	2502	2701	9.3	42.6	15.3	3.2	−19.1	7.8
57	Mondrian Tree 32MZ58	phalanx	2744	2497	2622	6.2	41.6	15.0	3.2	−19.8	5.8
58	Beacon Island 32MN234	molar	na	na	na	<1	na	na	na	na	na
59	Beacon Island 32MN234	molar	12396	12056	12229	3.7	41.7	14.9	3.3	−21.1	5.0
60	Beacon Island 32MN234	molar	12373	11845	12068	2.0	41.9	15.0	3.3	−21.2	5.7
61	Beacon Island 32MN234	molar	na	na	na	<1	na	na	na	na	na
62	Beacon Island 32MN234	M3	12547	12140	12431	4.2	41.4	15.2	3.2	−18.4	5.9
63	Beacon Island 32MN234	molar	na	na	na	<1	na	na	na	na	na
64	Mondrian Tree 32MZ58	phalanx	3825	3640	3705	5.6	40.3	14.3	3.3	−20.7	8.1
65	Brand Bison Kill 32SK201	horncore	691	661	675	8.7	45.6	16.7	3.2	−18.8	7.5
66	Rustad Site 32R1775	molar	8191	8042	8116	3.8	41.7	14.9	3.3	−16.2	8.1
67	Rustad Site 32R1775	M3	8178	8036	8107	5.1	40.5	14.7	3.2	−17.5	6.8
68	Rustad Site 32R1775	molar	8187	8042	8116	5.0	41.5	14.9	3.3	−16.2	7.8
97	Streifel Site 32ML903	phalanx	4984	3998	3913	10.5	42.4	15.2	3.3	−19.3	6.5
98	Streifel Site 32ML903	bone fragment	7932	7793	7862	3.8	42.3	14.9	3.3	−19.1	8.5
99	Streifel Site 32ML903	phalanx	na	na	na	<1	na	na	na	na	na
100	TRNP (bull)	M3	na	na	modern	13.3	42.4	15.6	3.2	−21.2	5.0
101	TRNP (bull)	molar	na	na	modern	16.9	42.9	15.5	3.2	−20.1	5.2
102	TRNP (cow)	M3	na	na	modern	17.4	41.9	15.1	3.2	−20.7	4.6
103	TRNP (cow)	M3	na	na	modern	15.8	42.7	15.6	3.2	−20.0	5.0
104	TRNP (cow)	molar	na	na	modern	20.4	42.3	15.5	3.2	−20.8	4.7

We determined that the age of ancient bison specimens range from the Late Pleistocene to the Late Holocene, 12,344 to 104 calibrated years before present (cal BP) with samples from the Late Pleistocene (~12.5 cal BP, n = 4), Early Holocene (11,700–8,200 cal BP, n = 1), Middle Holocene (8,200–4,200 cal BP, n = 7), and Late Holocene (4,200–100 cal BP, n = 43) (Table 2)^47^.

**Table 2:** Summary statistics and %C_4_ plants for bison bones and teeth within each episode.

Episode (cal BP)	Tissue	n	Mean δ^13^C ‰	SD δ^13^C ‰	δ^13^C ‰		Mean δ^15^N ‰	SD δ^15^N ‰	δ^15^N ‰		% C_4_
					low	high			low	high	
Modern	Dentin	5	−20.5	0.47	−21.1	−20.0	4.9	0.24	4.6	5.2	0
Late Holocene (4,200 – 100)	Dentin	25	−18.3	1.57	−20.5	−15.1	6.7	0.85	5.0	8.3	14
	Bone	18	−19.0	1.67	−21.1	−14.8	6.6	0.94	4.9	8.1	9
Middle Holocene (8,200 – 4,200)	Dentin	5	−16.8	0.82	−17.9	−16.2	8.2	0.96	6.8	9.3	24
	Bone	2	−16.9	3.18	−19.1	−14.6	6.8	2.47	5.0	8.5	24
Early Holocene (11,700 – 8,200)	Bone	1	−18.2	na	−18.2	−18.2	6.6	na	6.6	6.6	14
Late Pleistocene (12,500 – 11,700)	Dentin	4	−20.6	1.44	−21.5	−18.4	5.7	0.51	5.0	6.2	0

As previously stated, dentin and bone collagen represent different lengths of time in the bison’s life. The sampling method in this study for dentin likely captures less than a year^20^ while the collagen from bone samples describes the average over several years of the animal’s diet^16–18^. To compare the variation found in each tissue type, we conducted t-tests for the dentin and bone samples as a whole and also separated into temporal groups. We found no significant differences in the means or variation found in sample tissue types (Supplementary Figure S1), therefore, they are treated functionally the same for the purpose of this paper.

**Figure 2:**
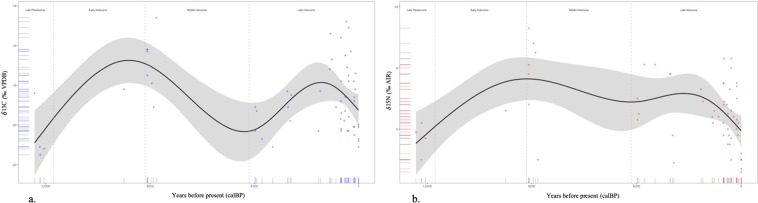
Estimated trendline of isotopic values over time for **(a**) carbon and (**b**) nitrogen. Smoothed functions were determined using generalized additive models with shaded areas representing 95% confidence intervals and points representing the data points. Dashed lines separate sub-epoch boundaries; Late Pleistocene – Early Holocene (11,700 ya), Early Holocene – Middle Holocene (8,200 ya), and Middle Holocene – Late Holocene (4,200 ya). Stable isotope measurements were determined on amino acid hydrolysate samples.

**Figure 3:**
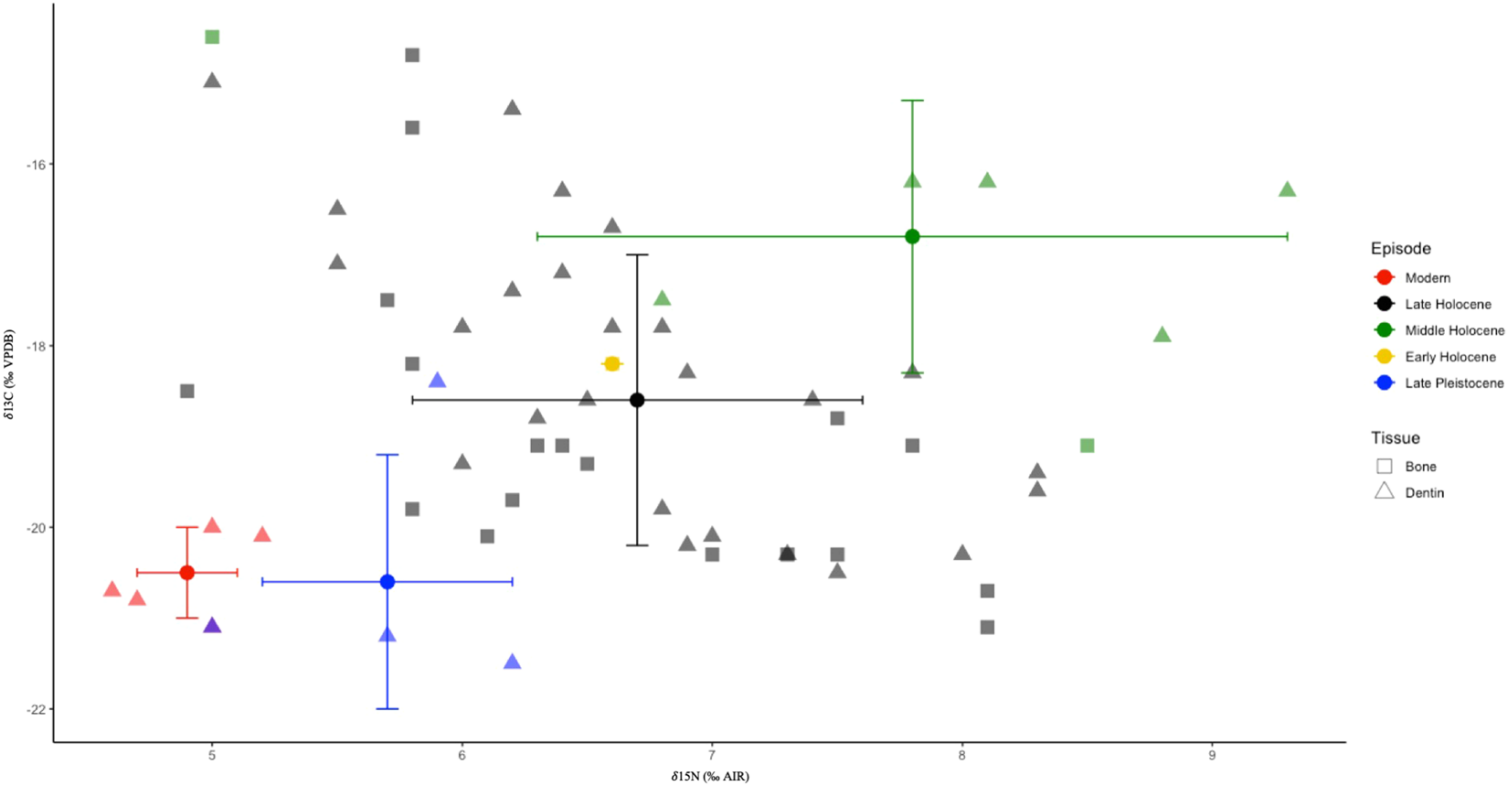
Plot of δ^13^C and δ^15^N values for bison by temporal episode. Bone samples are represented by squares and dentin samples are labeled with triangles. Means and standard deviations are plotted for temporal episodes. Stable isotope measurements were determined on amino acid hydrolysate samples.

**Figure 4:**
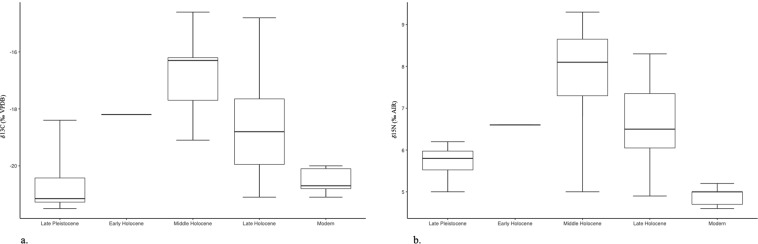
Box plots of (**a**) δ^13^C and (**b**) δ^15^N values measured for Late Pleistocene, Early Holocene, Middle Holocene, Late Holocene, and Modern bison. Boxes show the median and upper and lower quartiles while the whiskers show the range of values. Stable isotope measurements were determined on amino acid hydrolysate samples.

Generalized additive models (GAMs) identified significant changes in bison δ^13^C (y = −18.42, p = 0.0008) and δ^15^N (y = 6.49, p = 0.002) values over time (Fig. 2). Model fit values are included in Supplementary Table S2. Bison dentin δ^13^C values ranged from −21.5 to −15.1‰ and dentin δ^15^N ranged from 4.6 to 9.3‰. Bison bone δ^13^C values ranged from −21.1 to −14.6‰ and bone δ^15^N ranged from 4.9 to 8.5‰ (Table 2). Overall, we observed considerable variability among temporal episodes for isotope values δ^13^C and δ^15^N (Table 2, Figs 3, 4). Only one data point is categorized into the Early Holocene, however GAM models visualize the increase in bison’s carbon and nitrogen isotopic values throughout this sub-epoch (Fig. 2).

Modern bison had the least variation in carbon and nitrogen values and the lowest mean δ^15^N value, 4.9‰. The highest variation for both isotopes was seen in Middle Holocene bone samples. The highest mean δ^13^C and δ^15^N values, −16.8‰ and 8.2‰ respectively, were observed in Middle Holocene bison dentin. Late Pleistocene and modern bison dentin were the most depleted in ^13^C with a mean δ^13^C of −20.6‰ and −20.5‰, respectively (Table 2, Figs 3, 4).

Ratios of C_3_:C_4_ plants demonstrated that bison diet for each temporal episode predominately included C_3_ plants (Table 2). Late Pleistocene bison consumed entirely C_3_ vegetation with an increase in the consumption of C_4_ plants in the Early Holocene, a peak abundance of C_4_ material in the Middle Holocene and a small decrease again in the Late Holocene (Table 2, Fig. 2a). Modern bison in TRNP exhibited an entirely C_3_ diet, congruent with the vegetation found in the park^48^.

## Discussion

This study uses the archaeological record of bison in the Northern Plains to understand their evolutionary responses to environmental change and provide insight for best practices in bison conservation and management. Our analysis of isotopic values from bison remains spans the terminal phase of the Late Pleistocene to present, representing post-glacial changes in bison diet and vegetation associated with changing climate during the recent natural history of the species. The sample assemblage is contemporary with two bison population bottlenecks. The first bottleneck occurred during the terminal Pleistocene and the second in the late 19^th^ century when bison were nearly extirpated by humans^6,9^.

The Late Pleistocene in the Northern Great Plains is described as a time of sudden environmental change and a significantly wetter landscape after the recent retreat of the Laurentide ice sheet^49^. Pollen records from the study region indicate an abundance of *Picea* (evergreen) species and relatively low amounts of herbaceous plants (grasses and forbs)^49,50^. Late Pleistocene bison samples in the current study are derived from Beacon Island, a Paleoindian kill site in the Agate basin (Fig. 1). The concurrent stratigraphic layer at this site exhibits C_3_ dominated plant material^51^ and is congruent with the 100% C_3_ diet recorded in the δ^13^C of bison remains (Table 2). However, bison δ^15^N values from the Late Pleistocene appear surprisingly low considering environmental conditions in the Late Pleistocene were adverse enough to wipe out the majority of megafaunal species and facilitate bison’s first recorded population bottleneck^2,8^. Though low nitrogen values could be explained by a heavier dependence on nitrogen poor browse material in bison diet^17^. Substantial incorporation of browse is reported by other Late Pleistocene bison paleoecology studies in North America based on stable isotopes and bison dentition wearing patterns^33,52^. Additionally, more canopy cover from prominent evergreens would lower the abundance of nitrogen in plant material consumed^29^. However, we cannot ignore that this could also be an effect of small sample size from only bison dentin for this temporal period.

Evergreen forests south of the Laurentide ice sheet were rapidly succeeded by other vegetative communities in the transition between the Pleistocene and the Holocene^49,53,54^. By the Early Holocene, new deciduous forest south of the evergreens formed and bordered along riparian areas while grasslands spread throughout the open landscape^54^. As the Great Plains quickly became dominated by prairie^49,54^ bison migrated northward into the developing terrain and became plentiful at this time^53^. Yet only one data point from our sample assemblage falls within the Early Holocene boundaries. This single bison indicates an increase in C_4_ vegetation incorporated into diet (Table 2), consistent with rising temperatures and the presence of more C_4_ *Chloridoideae* grasses^51,55^. Additional evidence of an increase in C_4_ grasses are recorded in bison living during the Early Holocene within present day Yellowstone National Park and the state of Nebraska^40,56^. Nitrogen levels are higher than in Late Pleistocene bison despite an increase in effective moisture in the Early Holocene^55^, further supporting the idea that bison selected more browse material from low light areas in evergreen forests during the Late Pleistocene.

The Middle Holocene climate is summarized as highly variable with an overall shift towards warmer, drier conditions and patchiness of resources^1^. Herbaceous plants fluctuated throughout the Middle Holocene, alternating between *Poaceae* and *Ambrosia* (ragweed) communities, indicating frequent changes in precipitation^50,57^. Bison from this sub-epoch exhibit the highest mean values for carbon and nitrogen as well as large variation in bone samples (Table 2, Figs 3, 4). Although the two bone samples from the Middle Holocene (Bison 22 and 98) coincide in both time and space, they exhibit large isotopic differences (Table 1, Figs 1, 3). This could be representative of the vastly fluctuating climate or a remnant of different feeding strategies among bison sexes^37^. In any case, we observe the greatest amount of C_4_ vegetation in bison diet at this time for both tissue types (24%), indicating a trend towards longer and warmer growing seasons^39^. This is corroborated by bison isotopic values from the Eastern Great Plains during the Middle Holocene^11^.

Climatic conditions in the Northern Great Plains during the Late Holocene generally followed a cooling trend with increasing moisture up to modern day^26,55^. However, climate proxies provide evidence that severe arid conditions occurred at intervals throughout this time period^26,49^. We do not observe any obvious indicators of drought in bison samples from the Late Holocene but changes in vegetation type may lower the amount of nitrogen available in soils and dampen the signal of physiological stress^58^. A wide range of δ^13^C values and more C_4_ plant material is recorded in Late Holocene bison (Table 2, Figs 3, 4), suggesting diverse vegetation utilized and a continued shift towards extended growing seasons^39,41^. Pollen records indicate that *Poaceae* increased in abundance during the Late Holocene and the first appearance of *Salaginella densa* (spikemoss) is documented in the Northern Great Plains^50^. The ground cover provided by spikemoss and its ability to persist in dry conditions provides protection from erosion and forage to subsist on during lean winter months. Its expansion likely increased foraging capacity and contributed to the immense presence of bison on the prairie during the Late Holocene.

Coinciding with the environmental changes that took place during the Late Holocene is the rise of a more complex human ecosystem throughout North America. How humans influenced landscapes, bison behavior, and available food supplies during the Holocene are currently not well understood but research suggests they had an active role in the Great Plains ecosystem^59^. Bison’s past response to changing composition of habitat remains unclear but their ability to adapt and exploit a variety of resources is attributed to the species’ long-term survival over other megafauna.

Finally, we compared isotopic values of modern bison dentin from TRNP with ancient bison from the Late Pleistocene through the Late Holocene. We observe comparatively low variability in modern bison stable isotopes (Table 2, Figs 3, 4). TRNP bison have a more restricted rangeland than their ancient counterparts as well as a presumably shorter window for sample collection. Modern bison are depleted in ^13^C, similar to Late Pleistocene bison (Figs 3, 4), indicating a diet of 100% C_3_ plant material (Table 2) despite different climatic conditions experienced by the temporal groups. Nitrogen values are notably lower than in other bison groups (Table 2, Fig. 4), indicating no evidence of nutritional stress and adequate available moisture^27^. While low variation may be attributed to small sample size, other studies have found similar results in several living herds^25,39^. *Tieszen (1994)*^13^ showed that the Wind Cave National Park bison herd in South Dakota had a diet with more C_4_ plants but bison also contained a small amount of δ^13^C variability within the herd. The Catalina Island bison population also exhibits a comparable δ^13^C average and low variability^22^. Modern Yellowstone National Park bison exhibit low variability in δ^13^C values and a similar mean to the TRNP bison in this study despite their ability to cover much larger areas and complete substantial elevational migrations^37,60^. Whether this trend in low variability in modern bison diet is due to restricted rangelands and herd management practices or if it is a result of a narrowing in plasticity from the recent genetic bottleneck in bison history is still not clear. We would expect that if it were only due to the habitat restrictions imposed upon modern bison, we would observe more variability in Yellowstone herds.

Understanding the predecessors of present-day bison may unlock new views for reintroducing them more broadly to the North American landscape. These techniques are already influencing management decisions for European bison (*Bison bonasus*). European bison had to overcome similar environmental challenges as North American species during the terminal Pleistocene and are also predominately constricted to limited rangelands today^61^. Most habitat of modern European bison is forested but their morphological adaptations suggest they evolved in open grasslands^62^ and then moved into woodland areas as the forests expanded and pressure from humans increased^29^. Isotopic studies of the ancient Eurasian steppe bison (*Bison priscus*) are informing conservation strategies for their ecological successor, *Bison bonasus*^63^. Several studies have found that *Bison priscus* did rely heavily on grazing, with more browse incorporated over time, as woody vegetation became more accessible^52,61^. This information makes the introduction of European bison to more open grassland habitats a plausible strategy for large scale restoration and is an example of the value of conservation paleobiology for current species management.

The North American landscape has been transformed dramatically during the last 250 years, and with few exceptions, bison are no longer allowed to migrate or range widely in localities where they currently exist. Further, the extreme population bottleneck experienced by bison at the end of the 19^th^ century has left the species with only a microcosm of the genetic toolkit that it once wielded for adaptation. Thus, both the resiliency of the species and the landscape it once inhabited have been altered in a manner unprecedented since the last ice age. We may expect that genetically isolated and spatially confined herds will be the most challenged by environmental fluctuations^64^. Range expansion efforts such as opening of new state, federal, and tribal lands to bison and establishment of conservation herds on private lands are already underway, and bison range is currently expanding, but only at incrementally small amounts in comparison to the native range of the species.

Despite limitations imposed on present day herds, isotopic relationships identified here have provided a unique glimpse at paleoecology that is relevant to current management of the species. Managers would benefit from a spatially and temporally expanded study of bison isotopic profiles throughout the known historic range of the species. An evaluation of historic genetic diversity of the species may also reveal pre-extirpation global and local herd level values that can be used in parallel with isotopic data to illuminate ancient bison ecology and inform management. The common occurrence of bison remains in archaeological collections curated in facilities throughout North America makes this effort feasible and demonstrates the value of such collections to present day management of species and systems.

## Materials and Methods

### Sample assemblage

Ancient bison bone and tooth samples were collected from four North Dakota museum collections: North Dakota Heritage Center and State Museum (State Historical Society of North Dakota), Knife River Indian Villages National Historic Site, University of North Dakota’s Department of Anthropology, and University of North Dakota’s Biology Museum. Samples included in this project were collected as part of twenty-one previously excavated archaeological sites in North Dakota and one previously excavated site in northern South Dakota (Fig. 1). The context of the archaeological sites encompasses a large temporal scale from the Late Pleistocene throughout the Holocene. Holocene sites are associated with each North Dakota Native American cultural tradition as defined by long standing archaeological schema including Plains Village (AD 1200–1800’s), Plains Woodland (400 BC to AD 1700’s), Plains Archaic (5,500 BC to 400 BC) and Paleo-Indian (11,500 to 5,500 BC). To avoid repeat sampling of bison individuals, we selected the right 3rd molar whenever possible. In other cases, we selected the molars or large premolars from the right side of the jawbone. Specimens were also chosen from different stratigraphic layers in the archaeological site context. While every effort was made to not repeat samples in the assemblage, there is a minute chance that repeats were made in some cases.

The integrity of skeletal elements was observed under stereo microscopy, revealing well preserved tissues at surfaces and on cross section of bones and teeth. Preservation quality was determined by a clear delineation between cortical and spongy material with little discoloration, compact tooth dentin and opalescent enamel (Supplementary Fig. S3).

Modern bison teeth were obtained from animals (n = 5) culled for management purposes or those dying of natural causes within the North and South Units of TRNP. Bison range at TRNP includes two geographically separate units encompassing in total 28,542 ha the badlands of Western North Dakota. The Little Missouri River traverses park lands from south to north, and the landscape is characterized by stratified clay buttes capped with clinker and interspersed with lignite and fossils dating to the Paleocene Epoch. Annual precipitation is 38.1 cm, and vegetative communities include mixed grass prairie and sage (*Salvia* and *Artemisia* spp.) and other woody shrubs in uplands, cottonwood (*Populus deltoides*) galleries along riparian corridors, ash (*Fraxinus pennsylvanica*) groves in draws, and juniper (*Juniperus* spp.) stands along north facing slopes. Temperature varies widely, with means ranging from highs of approximately +29 °C in summer to lows of −18 °C in winter, with extremes sometimes ranging +43 to −43 °C.

At TRNP, bison forage alongside feral horses (*Equus caballus*), longhorn cattle (*Bos taurus*), elk (*Cervis elaphus*), pronghorn antelope (*Antilocapra americana*), mule deer (*Odocoileus hemionus*), white-tailed deer (*Odocoileus virginianus*), moose (*Alces alces*), sheep (*Ovis canadensis*), black-tailed prairie dogs (*Cynomys ludovicianus*), and other small mammals. Park lands are fenced, and in the absence of predators, bison are managed to prevent overgrazing through roundups, after which excess animals are transferred primarily to tribes. Herds have typically been allowed to range between 100–300 individuals in the North Unit and between 300–500 in the South Unit, in alignment with a forage allocation model and perceived social carrying capacity^48,65,66^.

Research was approved by the University of North Dakota (UND) Institutional Animal Care and Use Committee (protocol number 1511-5) and the UND Institutional Biosafety Committee (registration number IBC-201511-007). All methods and experiments were performed within the guidelines and regulations for the use of experimental animal specimens.

All archaeological samples were selected from human derived contexts. This provides potential to access both bison ecology and human selection processes that resulted in the culling and ultimate deposition of individual skeletal elements in the archaeological record. We acknowledge the human induced bias in our sample. Taking this bias into account allows us to access a record of bison population dynamics otherwise unavailable as a result of the late 19^th^ century population bottleneck.

### Sample preparation and isotope analysis

Sections of cortical bone or tooth dentin weighing 1–3 grams were cut from bison specimens using a band saw. Tooth dentin samples were taken in 0.5 to 1 inch contiguous pieces from dentin underlying enamel in the orientation of crown to cusp. Some tooth dentin portions included root material. Samples were then sent to the University of California Irvine (UCI) Keck Carbon Cycle Accelerator Mass Spectrometry Laboratory where they were decalcified in 1 N HCl and then gelatinized at 60 °C, pH of 2, and ultrafiltered to select for a high molecular weight fraction (>30 kDa). Aliquots of ultrafiltered collagen were measured on a Fisons NA1500NC elemental analyzer/Finnigan Delta Plus isotope ratio mass spectrometer to obtain δ^13^C and δ^15^N values at a precision of <0.1‰ and <0.2‰, respectively. Stable isotope measurements were determined on amino acid hydrolysate samples. Samples with C:N atomic ratios between 2.9 and 3.6 are indicative of well-preserved collagen^46,67^ and were then measured for ^14^C dating at the UCI Keck AMS facility using the methods outlined in *Lohse et al*.^11^. The error range for ^14^C ages (BP) in this study is ±15–35 years. All ^13^C to ^12^C ratios were reported relative to the Vienna PeeDee Belemnite (VPDB) standard and all ^15^N to ^14^N ratios are reported relative to the Ambient Inhalable Reservoir (AIR) standard.

### ^14^C Calibration and temporal episodes

^14^C ages were calibrated using OxCal 4.3^68^ and the IntCal13 curve^69^ for the Northern Hemisphere. All bison sample ages are reported in calibrated years before present (cal BP) and are within a 95% confidence interval. The median of the confidence interval was used as the sample date for separation into temporal episodes. All δ^13^C values dated before 1800 AD were adjusted by -1.5‰ to account for the reduction in atmospheric CO_2_ due to the increased burning of fossil fuels after the Industrial Revolution^13^.

The temporal range was split into episodes to allow comparisons between bison from the Late Pleistocene to modern. The episodes follow formal Holocene subdivisions recognized by the International Union of Geological Sciences (IUGS) and is based on data from Greenland ice cores, pollen records, lake sediments, and Global Stratotype Section and Points (GSSPs)^47^. The Pleistocene-Holocene boundary was marked at 11.7 thousand years ago. The Holocene was split into four episodes, Early Holocene (11.7 to 8.2 thousand years ago), Middle Holocene (8.2 to 4.2 thousand years ago), and Late Holocene (4.2 to 100 years ago)^47^. Modern bison are also considered an episode.

### δ^13^C and δ^15^N data analysis

We used generalized additive models (GAMs) to illustrate bison carbon and nitrogen isotopes over time with R statistical software and the package “mgcv”^70^. This method allows the estimation of isotope values between data points, providing a continuous view of bison isotopic fluctuations throughout the Holocene with a 95% confidence interval. GAMs were modeled with a gaussian distribution, an identity link function, and a smoothing parameter on time (k = 5).

The δ^13^C means of temporal episodes were also used to calculate the percentage of C_3_ and C_4_ grasses in bison using equation 1, modified from *Carlson et al. (2018)*^32^.

1 $$\begin{array}{rcl}{{\rm{C}}}_{{\rm{3}}}( \% ) & = & {[({\rm{\delta }}}^{{\rm{13}}}{{\rm{C}}}_{{\rm{collagen}}}-{\rm{6}}{{\rm{.3}}}_{{\rm{trophic}}{\rm{level}}{\rm{fractionation}}}-{{\rm{\delta }}}^{{\rm{13}}}{{\rm{C}}}_{{\rm{C4}}})/{({\rm{\delta }}}^{{\rm{13}}}{{\rm{C}}}_{{\rm{C3}}}-{{\rm{\delta }}}^{{\rm{13}}}{{\rm{C}}}_{{\rm{C4}}})]\times {\rm{100}}\\ {{\rm{C}}}_{{\rm{4}}}( \% ) & = & {\rm{100}}-{{\rm{C}}}_{{\rm{3}}}\,( \% )\end{array}$$

where δ^13^C_collagen_ includes a 6.3‰ adjustment for trophic level fractionation specific to bison^16,66^, δ^13^C _C4_ = −12.5‰ and δ^13^C_C3_ = −26.5‰.

## Supplementary information

Supplementary Materials

## Data Availability

All data generated for this study are included within this paper.
