# Supplementary material for: Isotopic paleoecology of Northern Great Plains bison during the Holocene
Source: Sci Rep. 2019 Nov 12;9:16637. doi: 10.1038/s41598-019-52873-4 (PMC6851189; doi:10.1038/s41598-019-52873-4)
Supplement: Supplementary file 1 — Supplementary Materials [file 41598_2019_52873_MOESM1_ESM.docx]

**Supplementary Materials**

**Isotopic paleoecology of Northern Great Plains bison during the Holocene**

Gaimi Davies^1^, Blake McCann^2^, Jay Sturdevant^3^, Fern Swenson^4^, Igor V. Ovchinnikov^1,5*^

^1^Department of Biology, University of North Dakota, Grand Forks, ND, USA. ^2^Resource Management, Theodore Roosevelt National Park, Medora, ND, USA. ^3^Midwest Archeological Center, National Park Service, Lincoln, NE, USA. ^4^Archaeology & Historic Preservation Division, State Historical Society of North Dakota, Bismarck, ND, USA. ^5^Forensic Science Program, University of North Dakota, Grand Forks, ND, USA. Correspondence and request for materials should be addressed to I.V.O. (email: igor.ovtchinnikov@email.und.edu)

| **Group** | δ**^13^C (‰ VPDB)** | δ**^15^N (‰ AIR)** |
| --- | --- | --- |
| **All Samples** | p = 0.76 | p = 0.91 |
| **Late Holocene** | p = 0.15 | p = 0.62 |
| **Middle Holocene** | p = 0.98 | p = 0.28 |

**Supplementary Table S1.** T-test p-value results for comparisons between tissue types for the entire sample set and for comparisons between tissue types within temporal groups.

| **Smoothed Function** | **k-index** | **p-value** |
| --- | --- | --- |
| **s(time) δ13C** | 1.01 | 0.46 |
| **s(time) δ15N** | 0.91 | 0.15 |

**Supplementary Table S2.** Generalized additive model checks. Models were fully converged after 6 iterations. Basis dimension (k) checking results for test of model fit with smoothing function (s) placed on time (i.e. age of sample).


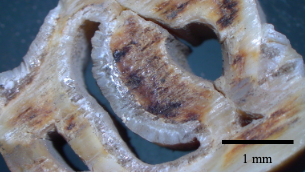

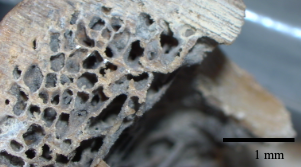


S3a. S3b.

**Supplementary Figure S3**. **a.** Bison specimen 13 (503 cal BP), cross section of a molar tooth. In good preservation, cementum is missing but dentin is intact and enamel has opalescent coloring. **b.** Bison specimen 31 (196 cal BP), cross section of a phalanx. Some surface discoloration and debris in inner structure, clear delineation between dense cortical and spongy tissue, spongy tissue is uniform, all indicative of moderate to good preservation.
